# Factors associated with job satisfaction among commune health workers: implications for human resource policies

**DOI:** 10.3402/gha.v6i0.18619

**Published:** 2013-01-30

**Authors:** Bach Xuan Tran, Hoang Van Minh, Nguyen Duc Hinh

**Affiliations:** Institute for Preventive Medicine and Public Health, Hanoi Medical University, Hanoi, Vietnam

**Keywords:** job satisfaction, human resource, commune health stations, health workers, Vietnam

## Abstract

**Background:**

Job satisfaction among health workers is an important indicator in assessing the performance and efficiency of health services.

**Objective:**

This study measured job satisfaction and determined associated factors among health workers in 38 commune health stations in an urban district and a rural district of Hanoi, Vietnam. A total of 252 health workers (36 medical doctors and 216 nurses and technicians; 74% female) were interviewed. A job satisfaction measure was developed using factor analysis, from which four dimensions emerged, namely ‘benefits and prospects,’ ‘facility and equipment,’ ‘performance,’ and ‘professionals.’

**Results:**

The results demonstrate that respondents were least satisfied with the following categories: salary and incentives (24.0%), benefit packages (25.1%), equipment (35.7%), and environment (41.8%). The average satisfaction score was moderate across four domains; it was the highest for ‘performance’ (66.6/100) and lowest for ‘facility and equipment’ (50.4/100). Tobit-censored regression models, constructed using stepwise selection, determined significant predictors of job satisfaction including age, areas of work and expertise, professional education, urban versus rural setting, and sufficient number of staff.

**Conclusion:**

The findings highlight the need to implement health policies that focus on incentives, working conditions, workloads, and personnel management at grassroots level.

The public health system in Vietnam is organized into three levels: central, provincial, and grassroots (1). Commune health stations (CHS), which serve as ‘gatekeepers’ of the public health system, provide primary health care, including curative and preventive services, and referrals to higher levels. CHS have an important role in managing 80% of the local demands for health care in a timely manner (2). Despite encouraging improvements in recent performance indicators, the Vietnam health system has been facing major challenges involving human resources for health care (1, 3). Previous studies have shown that grassroots health services are experiencing substantial shortages in staffing as well as competence (1, 3, 4). In addition, health workers are more likely to change jobs from lower to higher levels of the health system, and from rural to urban areas, where they may find higher income and more opportunities for professional development (3). Therefore, strengthening grassroots health services, with a focus on human resource development, is a key strategy to reduce overloads in the central levels as well as to relieve the social and economic burden of diseases in Vietnam.

**Policy recommendations**

- Needs-based human resource planning, health equipment, and infrastructure investments are necessary to improve the efficiency of commune health stations.
- Performance-based incentives and benefits for health workers working at the grassroots level should be encouraged.
- Continuing professional training and rearrangement of health staff can improve satisfaction among commune health workers.

In developed and developing countries, job satisfaction has been found to be a significant predictor of the quality and efficiency of the health systems (5–10). Generally, job satisfaction refers to the perception of health workers regarding various aspects of their work, such as atmosphere or conditions (5, 8, 9). Job satisfaction highly correlates with the retention of health workers in different levels of the health system (5, 11–13). Moreover, satisfied health workers also contribute to higher quality and better outcome of health services (5, 6, 14–16). Although measurements of job satisfaction may impact services management and human resource policy development, little is known about job satisfaction among Vietnamese commune health workers. A qualitative study by Witter et al. showed that overall satisfaction was associated with the recruitment and retention of doctors in rural Vietnam (3). In another study, Ladinsky et al. discussed the importance of ensuring human resources for the health care system, especially in the rural and low-income areas of Vietnam (2). Several studies have compared factors affecting job satisfaction among health care workers in developed and developing countries (6, 8, 10). Typical factors have included incentives and working conditions as well as interrelations and opportunities for health workers (3, 6). To support human resource policy development, we assessed job satisfaction and explored associated factors among grassroots health workers in an urban and a rural district of Hanoi, Vietnam.

## Methods

### Study setting

Hanoi has a population of about six million people living in 28 districts. We selected one urban district, Ha Dong, and one rural district, Thanh Oai, for the study. Located in central Hanoi, Ha Dong has approximately 200,000 citizens and 17 communes. Thanh Oai is a suburban area with 204,000 citizens and 21 communes. These two districts were selected for convenience of data collection; both represent a typical model of diseases found in rural and urban districts. All CHS in these two districts were studied.

### Study design and participants

We conducted a cross-sectional study of all health workers in 38 CHS in these two districts from June to December 2009. A total of 252 health workers voluntarily agreed to participate in the study.

### Data collection

Face-to-face interviews using structured questionnaires were conducted by trained senior medical students under the supervision of Hanoi Medical University faculty members.

### Measure and criteria

We collected information about individual characteristics of commune health workers, including sociodemographics, areas of expertise and employment, training and education, and length of service. We developed a standard measure to assess job satisfaction for the respondents. First, a systematic review of relevant English-language literature was conducted in order to compile criteria related to job satisfaction for Vietnam's health system. Second, we conducted focus group sessions with health managers, commune health workers, and experts in health management in order to identify factors that are important from various perspectives. Third, the list criteria was shortened based on each item's importance and relevance. Using a small sample, we conducted a brief trial to determine whether any cultural, language, or administration methods should be revisited. The final study included 15 questions on the following dimensions: 1) relationship with leaders; 2) relationship with colleagues; 3) salary and benefits; 4) training and future prospects; 5) facility; 6) current assigned tasks; and 7) appreciation of local people. The response options included a 5-point Likert scale: 1=very dissatisfied, 2=dissatisfied, 3=uncertain, 4=satisfied, and 5=very satisfied. Domain scores were estimated by averaging the score of all domain items and then linearly transforming the results to a (0; 100) scale. The higher scores indicated higher levels of job satisfaction in commune health workers.

### Statistical analysis

Statistical analysis was used to describe the characteristics of respondents. Exploratory factor analysis was applied to examine the construct validity of the job satisfaction measurements. Four factors were extracted by principle component analysis at an eigenvalue of ≥1.0, and the threshold was defined using the scree test. (The scree test assumes that important factors have a large variance, thus, it orders the factors by variance and then plots the variance against the factor number.) The threshold was defined where the eigenvalue curve flattened out (17). Orthogonal varimax rotation with Kaiser's normalization was used to reclassify the measure items in order to increase the interpretability of these factors. The cutoff point for factor loadings was set at 0.40. We had a cross-loading in one item and assigned it to the corresponding domain based on the nature of the questions and the overarching dimension. Internal consistency reliability of measurement was estimated using Cronbach's alpha. Cronbach's alpha is a function of the number of test items and the average intercorrelation among the items. This metric illustrates how closely related measured items are as a group. A Cronbach's of ≥0.7 indicates a reliable scale for group measurement.

Because domain scores ranged at (0; 100), they were left- and right-censored. Censoring from above and below the domain scores did not allow us to measure exactly the values that were higher or lower than the range thresholds. Therefore, Tobit-censored regression was used to determine factors associated with four domain scores of job satisfaction measurement. The Tobit model assumes that the distribution of data is censored normal and that the overall log-likelihood consists of two parts: the classical regression for the uncensored observations and the relevant probabilities that an observation is censored (18). In addition, the data generating process for censored and uncensored portions of the data are assumed to be similar. We used residuals to examine whether the normality and homoscedasticity assumptions were valid.

The candidate predictors of the model included individual characteristics, professional work, expertise, and training. The reduced model was constructed using stepwise forward model selection based on the log-likelihood ratio test at a *p* value<0.1, and it excluded variables at *p* values>0.2. The level of signification was set at a *p* value less than 0.05.

## Results

### Characteristics of participants

Respondent statistics are presented in Table 1. Health workers had served at their CHS for an average of 13.5 years; 26% were male; 14% had a medical doctor (MD) or higher specialized degree; and 34% worked in the clinical and treatment areas.

**Table 1:** Characteristics of commune health workers

	Total (*N*=252; 100%)	
	
	Mean	SD
Age	38.6	9.3
Working years	14.9	10.1
Working years at this CHS	13.5	9.2
Salary (1,000 dong/month)	1,810.1	607.2
	*N*	%
Male	65	26.0
Professional degree		
Specialized MD	6	2.4
MD	30	11.9
Nurses and technicians	216	85.7
Training place		
Hanoi	67	26.6
Ha Tay	128	50.8
Others	57	22.6
Areas of work		
Treatment	87	34.5
Paraclinical	9	3.6
Pharmacy	28	11.1
Preventive medicine	47	18.7
Management	35	13.9
Other	46	18.3
Areas of expertise		
General	128	51.8
Major specialty	56	22.7
Minor specialty	44	17.8
Others	19	7.7
Have job description	154	61.1

### Psychometric properties of the job satisfaction measure

Table 2 shows the construct validity and reliability of the job satisfaction measurement. In factor analysis, four main factors were selected that accounted for 59.2% of the variance, namely benefits and prospects, facility and equipment, performance, and professionals. The first factor, benefits and prospects, accounted for 33.3% of the variance, and all major factors had at least three items. The performance domain referred to the satisfaction of health workers with their current tasks, collaboration with other colleagues, and the appreciation of clients and local people for their performance. The professional domain referred to the satisfaction of health workers with their opportunities for acquiring technical knowledge, training, and coaching, as well as self-perceptions of their capacity and competency. Cronbach's alpha was good to excellent across domains, ranging at (0.64; 0.80) (Table 2).

**Table 2:** Factor loadings and reliability of the job satisfaction measurement

		Dimensions			
		
Measure items	% satisfied	Benefits and prospects	Facility and equipment	Performance	Professionals
Salary and incentive	24.0	0.69			
Benefit package	25.1	0.80			
Technical and medical equipment	35.7		0.79		
Environmental sanitation and hygiene	41.8		0.70		
Training materials, medical practice guidelines	50.4		0.62		
Material facilities	51.6		0.79		
Access to technical materials and information	53.9	0.49			0.52
Professional training and coaching	57.6				0.58
Current assigned tasks	58.2			0.73	
Support and interest from higher health managers	60.7	0.69			
Institutional development plan	63.8	0.59			
Support and interest from local authorities	67.3	0.67			
Self-capacity and competency	77.6				0.84
Appreciation of local people	80.7			0.68	
Solidarity and collaboration among colleagues	84.6			0.77	
Internal consistency reliability		0.80	0.79	0.69	0.64
Mean domain score (0–100)		51.6	50.4	66.6	61.0
SD		17.8	19.7	13.3	17.0

### Job satisfaction and predictors for commune health workers

The lowest proportion of respondents were satisfied or very satisfied with the following five criteria: salaries and incentives (24.0%), benefit packages (25.1%), equipment (35.7%), environment (41.8%), and guidelines (50.4%) (Table 2). Meanwhile, more than 80% of respondents were satisfied with their relationship with colleagues and with the appreciation of local people. The average domain score was moderate across four domains; it was the highest for performance (66.6) and the lowest for facility and equipment (50.4) (Table 2).

Table 3 displays factors associated with domain scores of job satisfaction among commune health workers. Compared to CHS with sufficient numbers of staff, health workers in understaffed CHS reported significantly lower domain scores in benefits and prospects, facility and equipment, and professionals. Expertise was a significant predictor of job satisfaction. We found that a higher level of professional education was associated with a lower satisfaction in facility and equipment. In addition, workers in minor specialties were less satisfied with their benefits and prospects than general practitioners. Also, in the facility and equipment dimension, significantly lower satisfaction was seen in the rural area among younger workers, among those completing in-service professional training, and among those who did curative work (versus paraclinical work). The level of satisfaction with performance was higher among those health workers with a job description and those who had more work experience.

**Table 3:** Factors associated with job satisfaction among commune health workers

	Benefits and prospects	Facility and equipment	Performance	Professionals
	
	Coef.	Coef.	Coef.	Coef.
Rural vs. urban	−4.0 (−9.5; 1.6)	−17.0 (−22.6; −11.4)**		
Age		0.8 (0.3; 1.3)**		0.2 (−0.0; 0.5)
Female vs. male	−7.5 (−12.9; −2.0)**			
Total working years		−0.4 (−0.9; 0.1)	0.2 (0.0−0.4)*	
Working years in the current CHS	0.2 (−0.1; 0.6)			
Salary grades	−3.2 (−6.8; 0.3)			
Professional education (specialized MD^a^)				
MD		17.3 (0.1; 34.5)*	−6.8 (−12.4; −1.2)*	
Technician		24.0 (8.3; 39.8)**		5.7 (−1.1; 12.6)
Areas of expertise (general^a^)				
Major specialty		4.4 (−1.5; 10.3)		
Minor specialty	−8.2 (−14.7; −1.8)*			
Others			5.7 (−0.7; 12.1)	6.4 (−2.0; 14.7)
Training place (Hanoi^a^)				
Ha Tay		−12.3 (−18.3; −6.4)**	−2.9 (−6.5; 0.6)	
Others		−6.7 (−14.0; 0.5)		
Current training (none^a^)				
In-service	−18.0 (−37.9; 1.8)	−23.1 (−43.8; −2.4)*		
Full time		9.6 (−2.3; 21.5)		
Have job description vs. none	−3.9 (−8.8; 0.9)		3.9 (0.3; 7.4)*	
Areas of work (curative)				
Para-clinical	14.7 (−1.0; 30.5)	21.5 (5.1; 37.8)*		
Pharmacy			−4.4 (−10.1; 1.4)	
Preventive medicine			2.8 (−1.5; 7.1)	
Number of staff				
Insufficient vs. sufficient	−10.9 (−16.5; −5.3)**	−9.7 (−15.5; −4.0)**		−7.6 (−12.3; −2.9)**
Constant	75.9 (64.4; 87.3)**	25.8 (3.9; 47.6)*	63.2 (59.1; 67.3)**	52.1 (39.6; 64.6)**

95% Confidence intervals in parentheses.

* Significant at 5%

** significant at 1%.

a Reference group.

## Discussion

We assessed job satisfaction and its predictors for grassroots health workers in 38 CHS in the Ha Dong and Thanh Oai districts of Hanoi City using a newly developed measure. We found a moderate level of satisfaction across four dimensions; however, lower scores were seen in the areas of benefits and prospects and facility and equipment than those for the areas of performance and professionals. Important predictors of job satisfaction among commune health workers included age, areas of work and expertise, professional education, urban versus rural setting, and the sufficiency of human resources.

Our findings are consistent with previous studies examining job satisfaction of health workers in both developed and developing worlds (7, 8, 12, 19–21). In resource-scarce settings, health workers were usually dissatisfied with the availability of equipment and supplies, facility infrastructure, and professional development (15, 19, 21, 22). This study found a low satisfaction with salary and incentives, but a relatively high satisfaction with employment prospects. Health workers at the grassroots level appreciated the support of higher health managers and local authorities, as well as the development plan of their CHS. This finding is comparable to an observation in Iran where commune health workers were satisfied with their assigned tasks and colleagues but were highly dissatisfied with salary and benefit packages (22). In other Vietnamese settings, doctors who worked in rural areas and who were satisfied with their current job reported their interest in ‘stable work and salary’ that confirms the importance not only of benefits but of political support and future prospects (3).

The low scores for benefits and prospects and facility and equipment suggest the need for improving facility infrastructure and incentives for commune health workers that in turn may improve job satisfaction. Although the Vietnamese government encourages MDs and specialists to work at the grassroots level, we found that those doing so were less satisfied with their working conditions. Given the fact that MDs are the ones responsible for general care and treatment, it is necessary to sufficiently equip them to perform their tasks. Adequate numbers of health workers are critical for meeting the demands for local health care. In Vietnam, the strength of CHS is determined by the total catchments area population. We found that 63.9% of health workers reported employment shortages in CHS, and this resulted in a significant level of job dissatisfaction. This suggests that health managers should revisit human resource policies for health care workers at the grassroots level.

There are some policy implications for strengthening human resources for the grassroots health care system in Vietnam. First, the number and type of health care staff in CHS should correlate with health care demand and local conditions rather than population size. Second, performance-based incentives and benefits for grassroots health workers should be improved (23). Local authorities should also mobilize resources from other sectors to enhance the infrastructure and working conditions of health stations. Public–private partnership can play an important role in responding to health care demands and in improving the quality of health care services (24). Finally, continuing professional training and health staff rearrangement could improve job satisfaction among commune health workers.

Standard procedures in measure development and validation improved the properties of measurement for this study. But while the sample size of these two Hanoi districts was sufficient for the comparison of urban and rural settings, this limited the generalizability of study findings.

In conclusion, the moderate level of job satisfaction among commune health workers highlights the need for policies that improve incentives, working conditions, workloads, and personnel management for the health system of Hanoi. Other settings in Vietnam might also benefit from these improvements.
